# Behavioral, blood, and magnetic resonance imaging biomarkers of experimental mild traumatic brain injury

**DOI:** 10.1038/srep28713

**Published:** 2016-06-28

**Authors:** David K. Wright, Jack Trezise, Alaa Kamnaksh, Ramsey Bekdash, Leigh A. Johnston, Roger Ordidge, Bridgette D. Semple, Andrew J. Gardner, Peter Stanwell, Terence J. O’Brien, Denes V. Agoston, Sandy R. Shultz

**Affiliations:** 1 Anatomy and Neuroscience, The University of Melbourne, Parkville, VIC, 3010, Australia; 2 The Florey Institute of Neuroscience and Mental Health, Parkville, VIC, 3052, Australia; 3 Department of Medicine, The Royal Melbourne Hospital, The University of Melbourne, Parkville, VIC, 3050, Australia; 4 Department of Anatomy, Physiology, and Genetics, Uniformed Services University of the Health Sciences, Bethesda, MD, 20814, USA; 5 Department of Electrical and Electronic Engineering, The University of Melbourne, Parkville, VIC, 3010, Australia; 6 Centre for Stroke and Brain Injury, School of Medicine and Public Health, The University of Newcastle, Callaghan, NSW, 2308, Australia; 7 School of Health Sciences, The University of Newcastle, Callaghan, NSW, 2308, Australia

## Abstract

Repeated mild traumatic brain injuries (mTBI) may lead to serious neurological consequences, especially if re-injury occurs within the period of increased cerebral vulnerability (ICV) triggered by the initial insult. MRI and blood proteomics might provide objective measures of pathophysiological changes in mTBI, and indicate when the brain is no longer in a state of ICV. This study assessed behavioral, MRI, and blood-based markers in a rat model of mTBI. Rats were given a sham or mild fluid percussion injury (mFPI), and behavioral testing, MRI, and blood collections were conducted up to 30 days post-injury. There were cognitive impairments for three days post-mFPI, before normalizing by day 5 post-injury. In contrast, advanced MRI (i.e., tractography) and blood proteomics (i.e., vascular endothelial growth factor) detected a number of abnormalities, some of which were still present 30 days post-mFPI. These findings suggest that MRI and blood proteomics are sensitive measures of the molecular and subtle structural changes following mTBI. Of particular significance, this study identified novel tractography measures that are able to detect mTBI and may be more sensitive than traditional diffusion-tensor measures. Furthermore, the blood and MRI findings may have important implications in understanding ICV and are translatable to the clinical setting.

A mild traumatic brain injury (mTBI), often referred to as a concussion, rarely has lasting effects and is often presumed to cause only transient disturbances to brain function^1^,^2^,^3^,^4^,^5^. However, repeated mTBIs, particularly those occurring in the sports and military settings, have been associated with cumulative and chronic neurological impairments^6^,^7^,^8^, and the development of neurodegenerative diseases such as chronic traumatic encephalopathy (CTE)^6^,^7^,^8^,^9^. There is evidence that these long-term adverse effects of repeated mTBIs are in part due to the recurring insults occurring before the brain has recovered from the initial mTBI and is in a period of increased cerebral vulnerability (ICV)^5^,^10^,^11^. There is increasing evidence that mTBI triggers complex biological changes including inflammatory, metabolic, neuronal, vascular and axonal abnormalities^1^,^2^,^3^,^4^. It is believed that such changes are responsible for ICV and therefore, the identification of reliable markers that indicate when the brain is no longer in a state of ICV might allow them to be used to guide medical decisions.

The current clinical management of mTBI is largely guided by the presence or absence of neuropsychological symptoms, and typically evaluated by subjective and/or self-reported methods^2^,^3^,^4^. Symptoms may include physical, cognitive, co-ordination, emotional, and sleep abnormalities^2^,^3^,^4^. The onset of symptoms, although typically rapid, can take minutes or hours to occur, and symptoms are usually mild, or may even go unrecognized^2^,^3^,^4^,^12^,^13^. Recovery is determined to have occurred after all post-injury symptoms have resolved, at which point patients are commonly cleared to return to pre-injury activity^2^,^3^,^4^,^12^. However, there is now evidence that the resolution of symptoms might not accurately indicate that the brain has recovered from the neuropathophysiological changes induced by mTBI^1^,^2^,^3^,^4^,^5^,^10^.

Considering the possible long-term consequences of repetitive mTBIs, and the limitations of current mTBI management approaches, research is required to guide and facilitate more informed medical decisions pertaining to return to pre-injury activity. In particular, it is critical that objective markers sensitive to the brain’s changes and recovery after an mTBI are identified. Magnetic resonance imaging (MRI) is a non-invasive and common clinical tool that may be capable of providing objective and quantitative indicators of mTBI pathophysiology to help guide clinical management^1^,^2^,^3^,^4^,^5^,^11^. Although conventional structural MRI is often unable to identify pathological changes due to the absence of macroscopic changes^1^, initial studies applying advanced MRI techniques are gaining prominence as potentially highly sensitive indicators of mTBI. In particular, there is growing evidence that diffusion weighted imaging (DWI) and proton magnetic resonance spectroscopy (^1^H-MRS) may be sensitive to the subtle pathophysiological changes that occur in the mildly injured brain^14^,^15^,^16^,^17^,^18^. Blood-based biomarkers also hold great promise in the mTBI field, as there are several candidate protein biomarkers that may be indicative of neuronal and glial cell loss, metabolic abnormalities, vascular changes, neuroinflammation, axonal injury, and other pathophysiological mechanisms associated with mTBI^11^,^19^,^20^,^21^,^22^,^23^,^24^,^25^,^26^,^27^,^28^.

Although these initial MRI and blood-based protein findings demonstrate the potential of these methods to provide insight into brain abnormalities post-mTBI, more detailed studies are required to characterize and validate these methods as well as correlate the microstructural and molecular changes to the commonly used neurobehavioral outcomes. Animal models allow for the control of confounding factors, as well as the rigorous investigation of biomarkers. Previous studies from our laboratory, and others, report that a mild fluid percussion injury (mFPI) in the rat induces transient behavioral and pathophysiological changes that occur in the absence of significant neuronal loss or structural brain damage^29^,^30^,^31^, which is consistent with what may occur after a single mTBI in humans^2^,^3^,^4^. Therefore, here we employed the mFPI model in conjunction with serial multi-modal MRI, blood-based proteomics, and behavioral analyses in order to assess the ability of these methods to detect changes and estimate recovery after experimental mTBI.

## Methods

### Subjects

46 male Long-Evans rats were purchased from Monash animal research services (Melbourne, Australia). All rats were 8–12 weeks of age, weighed 250–300 g, and were experimentally naïve prior to surgical procedures. After surgery, rats were housed individually under a 12 h:12 h light/dark cycle with *ad libitum* access to water and food for the duration of the study. All experimental procedures were approved by The University of Melbourne and The Florey Institute of Neuroscience and Mental Health animal ethics committees, and complied with the guidelines of the Australian Code of Practice for the Care and Use of Animals for Scientific Purposes.

### Mild lateral fluid percussion injury (mFPI)

All of the surgical procedures used were based on standard protocols as previously described and used by our group^29^,^30^. Briefly, rats were placed in a sealed Plexiglas box into which 4% Isoflurane and 2 L/min oxygen flow was introduced for anesthesia. Rats were then placed in a stereotaxic frame via ear bars, with anesthesia maintained at 2% isoflurane and 1 L/min oxygen, and given a subcutaneous injection of analgesic (carprofen 5 mg/kg). A craniotomy (5 mm diameter) was performed (anterior/posterior −3.0 mm, medial/lateral 4.0 mm relative to Bregma) to expose the intact dura and a hollow injury cap was affixed to the skull over the craniotomy window. Rats were then attached to the fluid percussion device via the headcap. At the first sign of hind paw withdrawal to a pinch test, rats received a fluid percussion pulse of 1–1.5 atm before being disconnected from the device. Acute injury measures were monitored and included: duration of apnea, defined as the time from injury to the return of spontaneous breathing (sham/mFPI = 0/0 s); loss of consciousness, defined as the time to hind limb withdrawal to a pinch test (sham/mFPI = 0/1.9 ± 0.9 s); and time to self-righting, defined as the time from injury to return of an upright position (sham/mFPI = 82.8 ± 4.0/149.3 ± 9.2 s, *p* < 0.05). The protocol was identical for sham injuries but no fluid percussion pulse was given.

### Design

After their assigned sham or mFPI injury, rats were randomly allocated to separate behavior (sham = 8; mFPI = 8) or MRI (sham = 10; mFPI = 10) cohorts. The behavior cohort underwent behavioral testing on days 1 (D1), 3 (D3), 5 (D5), and 7 (D7) post-injury before being euthanized on D7 for blood collection. The MRI cohort underwent MRI at D1, D3, D5, D7 and 30 days post-injury (D30) before being euthanized on D30 for blood collection. Note that the MRI cohort also underwent behavioral testing on the day prior to their D30 MRI. This study design allowed us to maximize the amount of data collected on corresponding post-injury days, and also avoided the confounding factor of anesthesia (required for MRI) on behavioral outcomes. An additional 10 rats (sham = 5; mFPI = 5) were euthanized at D1 for blood analysis. Thus, blood samples were available for D1, D7 and D30 post-injury (5/group/day).

### Behavioral Testing

All behavioral testing was conducted by a researcher blinded to experimental conditions. Cognitive function was assessed using the water maze^29^,^30^. The water maze apparatus consisted of a circular tank (163 cm in diameter) filled with water (29 ± 1 °C), and a hidden acrylic escape platform (10 cm diameter) submerged 2 cm below the water surface in one of the quadrants of the pool. Unique visual cues were positioned at the north, south, east and west quadrants. Both the position of the escape platform, and the visual clues, were randomly assigned each day to maintain novelty throughout the course of the experiment. Rats completed four trials per testing day, with the entry position randomized between north, south, east and west for each trial. If the rat failed to find the platform in the allotted minute, then they were placed on the platform for 15 s. Ethovision behavioural tracking software analyzed video from an overhead camera positioned at the center of the tank. Search time and direct and circle swims (cognition), as well as swim speed (locomotion), were recorded for each trial.

Sensorimotor function was assessed using a 1 m long, 2 cm wide elevated wooden beam as previously described^29^,^30^. Baseline training was performed prior to surgery and consisted of five successful trials on a 4 cm wide beam, followed by five successful trials on a 2 cm wide beam. On each day of testing, rats completed ten trials on the 2 cm wide beam with the time taken to traverse the beam, as well as the numbers of slips and falls, recorded. The maximum time allowed per trial was 60 s, which was also assigned to rats that fell.

As previously described^29^,^30^, anxiety-like behavior was assessed using an elevated plus maze. The maze consists of both open and closed arms, 110 cm long and 12 cm wide, perpendicular to each other, and dividing each in half to form a cross. 50 cm high walls enclosed the closed arms while the open arms contained no walls. A single 5 minute trial was performed on each day of testing with the rat initially positioned at the center of the cross facing an open arm. Ethovision behavioural tracking software analyzed video from an overhead camera positioned at the center of the cross. The percentage of time spent within the open arm (anxiety), as well as the total number of entries into the closed and open arms (locomotion), were calculated. An entry was only completed once all four paws had entered one of the arms.

Locomotor activity and anxiety-like behavior were assessed using a circular open field (diameter 100 cm, 40 cm high wall) as previously described^29^,^30^. Ethovision behavioural tracking software (Noldus, Netherlands) analyzed video from an overhead camera positioned at the center of the field. A single 5 minute trial was performed on each day of testing with the rat positioned in the center of the field. The total distance travelled (locomotion), as well as the number of entries into and time spent within the center 66 cm diameter area of the field (anxiety), were recorded.

### MRI Acquisition

MRI scanning was performed on D1, D3, D5, D7 and D30 using a 4.7 T Bruker Avance III scanner with 30 cm horizontal bore. The magnet was fitted with a BGA12S2 actively shielded gradient set capable of 440 mT/m and actively decoupled volume transmit and 4-channel surface receive coils were used for imaging. Rats were anesthetized in a clear plastic box with 5% Isoflurane in a 1:1 mixture of medical grade air and oxygen. After loss of consciousness, rats were positioned supinely on a purpose-built rat cradle with stereotactic fixation and a nose cone positioned over the rat’s snout to maintain anesthesia with approximately 2% Isoflurane in a 1:1 mixture of medical grade air and oxygen. Body temperature was maintained throughout the experiment with a hot water circulation system built into the cradle. Arterial oxygen saturation, heart and breath rates and pulse distention were monitored with a MouseOx^TM^ pulse oximeter (STARR Life Sciences Corp. Pittsburgh PA, USA) and anesthesia adjusted accordingly.

The scanning protocol consisted of a 3-plane localizer sequence followed by multi-slice axial, coronal and sagittal scout images to accurately determine the position of the rat brain. A multi-echo T_2_^*^-weighted image was acquired using a 2D gradient echo sequence with the following imaging parameters: repetition time (TR) = 4.4 s; 8 echoes with the first echo at 7.5 ms and an echo spacing of 7.5 ms; field of view (FOV) = 28.8 × 28.8 mm^2^; matrix size = 160 × 160; number of slices = 64; and slice thickness = 180 μm.

Diffusion-weighted imaging (DWI) was performed using a 2D echo planar, spin-echo sequence with the following imaging parameters: TR = 6 s; echo time (TE) = 35 ms; FOV = 25.6 × 25.6 mm^2^; matrix size = 128 × 128; number of slices = 24; and slice thickness = 600 μm. Diffusion weighting was performed with diffusion duration (δ) = 3.5 ms, diffusion gradient separation (Δ) = 14 ms and b-value = 1200 s/mm^2^ in 81 non-collinear directions with 8 non-diffusion-weighted (b0) images.

Point resolved spectroscopy (PRESS) was acquired in the ipsilateral cortex with VAPOR water suppression and outer volume saturation. Acquisition parameters were: TR = 2.5 s; TE = 20 ms; number of excitations (NEX) = 700; spectral width = 6 ppm; number of points = 2048; offset = −2 ppm; and voxel size = 2 × 4 × 5 mm^3^. Non-water suppressed spectra were also acquired for eddy current correction and water scaling for metabolite quantification.

### MRI Analysis

#### T_2_^*^-weighted Imaging

A study-specific T_2_^*^-weighted template image was created by first generating templates for each cohort at each time point using Advanced Normalization Tools (ANTs, http://stnava.github.io/ANTs/). ANTs derives an optimal template based on symmetric normalization unbiased by an individual subject’s topographical idiosyncrasy^32^. The resulting templates were then combined, again with ANTs, to create the study-specific template. T_2_^*^-weighted images for each rat were then registered to the study-specific template (T_2_^*^-to-template) using standard symmetric normalization (SyN), a diffeomorphic algorithm included in ANTs^32^.

Eight *a-priori* regions of interest (ROIs) were traced using FSLView, a component of FMRIB’s Software Library (FSL, www.fmrib.ox.ac.uk/fsl), on the study-specific T_2_^*^-weighted template. Tracing started at the anterior tip of field CA3 of the hippocampus, approximately 1.72 mm posterior to bregma and continued for 16 slices. ROIs included the ipsilateral and contralateral cortex, hippocampus, corpus callosum and ventricles. The ROIs were registered to subject space using the inverse T_2_^*^-to-template diffeomorphisms and the total volumes for each structure were calculated using MATLAB (The MathWorks, Natick, MA, USA).

#### Diffusion-Weighted Imaging

Diffusion tensor images (DTI) were calculated using DTIFit, part of the FMRIB Diffusion Toolbox (FDT, part of FSL) and registered to a study-specific DTI template constructed using DTI-TK (http://dti-tk.sourceforge.net). DTI-TK uses the local fibre orientations to guide the alignment of white-matter tracts, which has been shown to result in improved registration over standard fractional anisotropy (FA)-based approaches^33^. Templates for each cohort at each time point were then used to generate a study template. Each rat’s DTI was warped directly to the study template (DTI-to-template) and resampled to have a resolution of 0.1 × 0.1 × 0.3 mm^3^.

The ipsilateral and contralateral cortex, hippocampus and corpus callosum were traced on the study template using FSLview for *a-priori* ROI-based analyses. Tracing started at approximately −1.8 mm bregma and continued for 10 slices. Template ROIs were warped back to individual space using the inverse DTI-to-template diffeomorphisms. For each ROI, the median of FA, radial diffusivity (RD), axial diffusivity (AD) and trace (TR) were calculated using MATLAB.

#### Tractography

DWI preprocessing for tractography was performed as described previously^34^. Briefly, spatial intensity inhomogeneity was corrected by estimating the bias field across the mean b0 image and images were normalized by the contralateral white matter. The fibre orientation distribution (FOD) within each voxel was estimated using constrained spherical deconvolution, with a group average single-fibre response function, to a maximum harmonic degree of 6.

Whole brain tractograms of 2 million streamlines were generated using the iFOD2 tractography algorithm, part of the MRtrix package (www.brain.org.au/software)^35^. Tracking was performed with the following parameters: FOD amplitude for initiating tracks = 0.2; FOD termination threshold = 0.1; FOD samples per step = 4; step size = 100 μm; minimum track length = 200 μm; maximum angle between steps = 22.5°.

**Figure 1:**
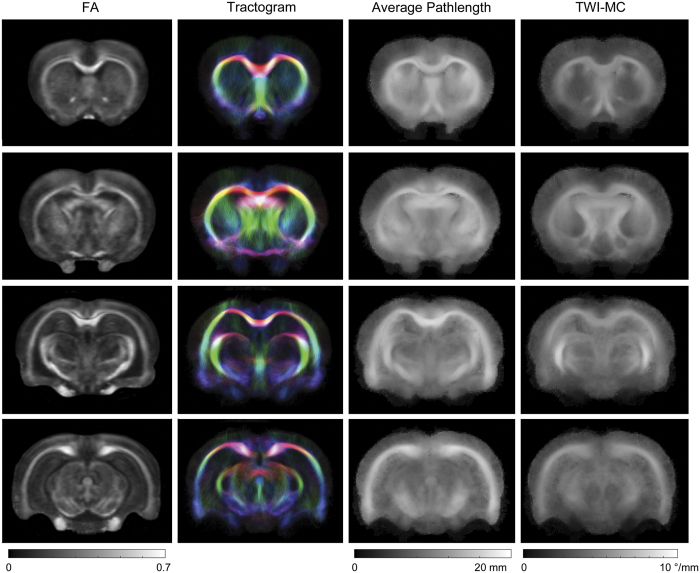
Tract-weighted imaging provides additional contrast to traditional diffusion tensor imaging. Example FA, tractogram, average pathlength and TWI-MC template images for sham rats at D30. Tractogram images were generated with one million streamlines, colour-encoded according to orientation: red, lateral/medial; green, superior/inferior; blue, anterior/posterior. Average pathlength and TWI-MC images calculated from the tractograms show novel contrast compared to the FA map. TWI-MC = track-weighted mean curvature.

Tractograms were registered to the final DTI template using the DTI-to-template diffeomorphisms ensuring that both the length and spatial location of the streamlines were normalized^36^. The tractograms were then used to generate two tract-weighted images: average pathlength map, representing the average length of tracks passing through each voxel^36^; and track-weighted curvature map, representing the average curvature of all tracks passing through each voxel (TWI-MC). Tractogram derived average pathlength and TWI-MC images show novel contrast not observed in diffusion tensor images such as FA (see Fig. 1). As with the tensor measures, the median of each was calculated for the *a-priori* ROIs.

#### Magnetic Resonance Spectroscopy

MRS data was processed using LCModel with a metabolite basis set matched to field strength and TE. The combined absolute concentrations of glutamate + glutamine (Glu+Gln), creatine + phosphocreatine (Cr+PCr), N-acetylaspartate + N-acetylaspartylglutamate (NAA+NAAG), glycerophosphocholine + phosphocholine (GPC+PCh) as well as the single peaks *myo*-inositol (Ins) and glutathione (GSH), were quantified. Maximum uncertainty in the concentrations (Cramér-Rao lower bounds) was 5% for the combined concentrations and 10% for Ins and GSH.

### Plasma Analysis

#### Blood collection and plasma separation

Following the completion of experiments on D1, D7, or D30 (see Design above), rats were deeply anaesthetized by an intraperitoneal injection of 0.5 ml sodium pentobarbital (Lethabarb, Virbac, Australia). The chest cavity was then opened and blood was collected into a BD Vacutainer K2 EDTA (K2E) Plus Blood collection tube. The tubes were then centrifuged at 6,000 g for 15 min at room temperature, and plasma was pipetted into 0.5 ml aliquots, flash-frozen in liquid nitrogen, and stored at −80 °C until use.

#### Proteomics

Plasma levels of ceruloplasmin, neurofilament heavy chain (NF-H), tau protein, vascular endothelial growth factor (VEGF), 4-hydroxynonenal Michael adducts (4-HNE), neuron-specific enolase (NSE), glial fibrillary acidic protein (GFAP), and S100 calcium binding protein β subunit (S100β) were assayed using reverse phase protein microarray (RPPM). Sample preparation, printing, scanning, and data analysis for RPPM were performed as described previously^19^,^20^,^21^,^22^,^23^,^37^,^38^,^39^,^40^. Frozen plasma samples were thawed on ice and diluted 1:10 with Dilution Buffer (3 parts Lysis Buffer [TPER, 10% Glycerol, 1× HALT] and 1 part 4× SDS Sample Buffer [35% Glycerol, 0.8% SDS, 10× TBS, 10× TCEP, 1× HALT, 0.0035% NaN_3_]). Samples were then transferred to a JANUS Varispan Integrator and Expanded Platform Workstation (PerkinElmer, Waltham, MA) to perform 1:1 serial dilutions using Dilution Buffer in 384-well microarray plates (product no. X7022; Molecular Devices, Sunnyvale, CA). An equal amount of 2× PBS Buffer (80% Glycerol, 2× PBS) was added to the volume of liquid in each well. The microarray plates were subsequently transferred into an Aushon 2470 Arrayer (Aushon Biosystems, Billerica, MA) where plasma samples were printed onto ONCYTE^®^ AVID nitrocellulose film slides (product no. 305177; Grace Bio-Labs, Bend, OR). The Aushon 2470 Arrayer was set up with 16 pins and programmed for 2 depositions per spot. The spot diameter was set to 250 nm with spacing between dots at 500 nm on the x-axis and 375 nm on the y-axis. Wash time was set to 2 s without delays.

#### Immunochemical detection

After overnight desiccation at 4 °C, the slides were blocked with a solution of 1× TBS, 5% non-fat dry milk, and 0.1% Tween-20. Slides were then incubated with the primary antibody solutions and a cover slip (product no. 25x 60I-M-5439-001-LS; mSeries LifterSlip; Thermo Fisher Scientific, Waltham, MA) overnight at 4 °C. All primary antibodies were pretested for specificity by Western blot analysis using positive controls and neutralizing peptides when available^37^. The primary antibodies were diluted in antibody incubation buffer (0.1% BSA, EDTA-free Halt Protease and Phosphatase Inhibitor Cocktail [Thermo Fisher Scientific], 1× TBS, and 0.5% Tween-20) and used in the following dilutions: ceruloplasmin (1:20; Santa Cruz Biotechnology, sc-21240), NF-H (1:100; Sigma–Aldrich, N4142), tau (1:100; Cell Signaling Technology, 4019), VEGF (1:50; Abcam, ab53465), 4-HNE (1:1000; EMD Millipore, 393207), NSE (1:50; Abcam, ab53025), GFAP (1:20; Abcam, ab48050), and S100β (1:20; Abcam, ab41548).

The following day, slides were washed three times with TBST and then incubated with the appropriate secondary antibody solutions for 1 h at room temperature. The secondary antibodies Alexa Fluor^®^ 790 goat anti-rabbit (catalog no. A-11369), 680 rabbit anti-goat (catalog no. A-21088), and 680 goat anti-mouse (catalog no. A-21058) (Invitrogen, Eugene, OR) were used at a 1:20,000 dilution. After three thorough washes with TBST followed by a single wash with 1× TBS, the slides were air dried and subsequently scanned in an Innopsys InnoScan 710 IR microarray scanner (Innopsys, Carbonne, France).

#### Data acquisition and analysis

Scanner fluorescence data were imported into a Microsoft Excel-based bioinformatics program. After correcting for local background noise, points indiscernible from background were excluded (SNR < 2, Net Fluorescence < 10). Net intensity vs. dilution was plotted on a log-log scale. A five-parameter logistic (5PL) master curve was fitted to the slide. Using this master curve, outliers were excluded using the False Discovery Rate method (Q = 0.01) and the master curve is further refined. Each local block of samples is fit individually, using master curve values as the initial conditions for the local curve fit. The asymptotic maximum and minimum, Hill-Slope, and asymmetry constant were shared between samples. The slope of the linear portion of the logistic curve was calculated and the line extrapolated back to zero (i.e., the y-intercept), assessing the amount of protein expressed. Data (y-intercept values) are presented as the mean ± S.E.M.

### Statistical analysis

Between group differences in behavior, MRI and plasma protein levels were analyzed with independent t-tests using SPSS Statistics 22 software (IBM, New York, NY). Statistical significance was set at *p* < 0.05 in all cases.

## Results

### mFPI induces transient cognitive deficits in the water maze

**Figure 2:**
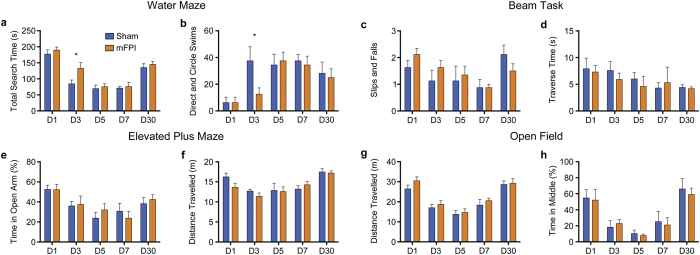
mFPI results in transient cognitive deficits. Rats given an mFPI had longer total search times (**a**) and fewer direct and circle swims (**b**) during water maze testing on D3. There were no group differences on Beam Task (**c,d**), elevated-plus maze (**e,f**) or open field (**g,h**). Mean ± s.e.m., *mFPI group significantly different than sham group, *p* < 0.05.

Rats were tested in the water maze to assess cognitive function. Rats given an mFPI showed significantly longer search times in the water maze on D3 compared to sham-injured rats (t = 2.215, *p* = 0.044, Fig. 2a). Consistent with these findings, mFPI rats also performed fewer direct and circle swims compared to sham-injured rats on D3 (t = 2.160, *p* = 0.047, Fig. 2b). Notably, by D5 there were no significant differences on either of these measures of cognitive ability between mFPI and sham groups. There were no significant differences on the measure of swim speed in the water maze on any of the days, suggesting that motor abnormalities were not a confounding factor in cognitive outcomes. There were no significant differences (all *p* > 0.05) between mFPI and sham rats on any of the beam task (sensorimotor function; Fig. 2c,d), elevated-plus maze measures (anxiety-like behavior; Fig. 2e,f) or open field (locomotion, anxiety-like behavior; Fig. 2g,h).

### mFPI does not result in macroscopic brain damage

**Figure 3:**
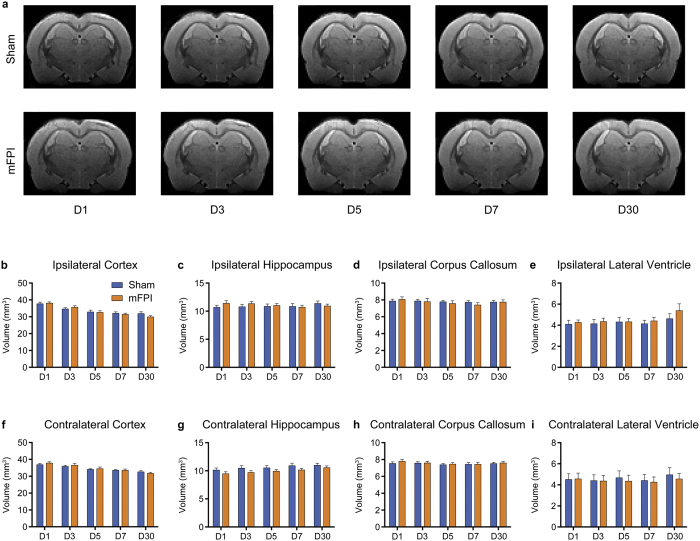
mFPI did not result in significant volumetric changes. (**a**) T_2_^*^-weighted template images for sham (top row) and mFPI (bottom row) groups. There were no significant differences observed in any of the eight *a-priori* ROIs (**b–i**). Mean ± s.e.m.

To assess for any overt structural brain damage we conducted a volumetric analysis on T_2_^*^-weighted images. As shown in Fig. 3, mFPI did not result in significant volumetric changes to any of the eight ROIs, including the ipsilateral and contralateral cortex, corpus callosum, hippocampus, and lateral ventricles (all *p* > 0.05).

### mFPI induces transient ^1^H-MRS abnormalities

**Figure 4:**
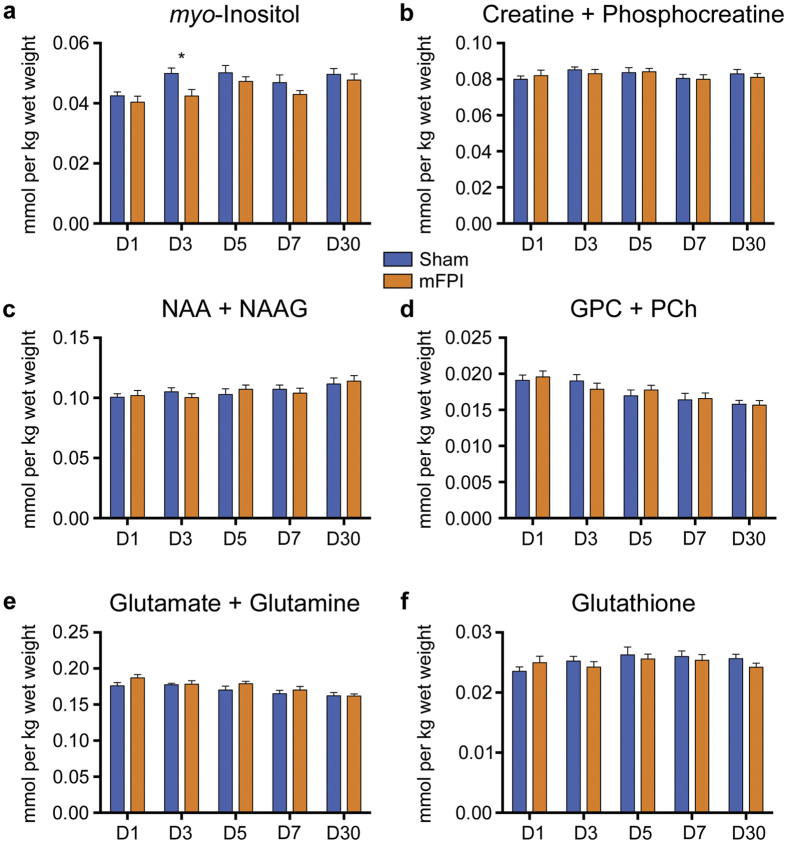
mFPI induces transient ^1^H-MRS abnormalities. mFPI rats had significantly decreased myo-inositol at D3 compared to sham animals (**a**). There were no significant differences observed in any other metabolites (**b–f**). NAA+NAAG = N-acetylaspartate + N-acetylaspartylglutamate; GPC+PCh = glycerophosphocholine + phosphocholine. Mean ± s.e.m., *mFPI group significantly different than sham group, *p* < 0.05.

^1^H-MRS analyses revealed that mFPI rats had significantly lower Ins in the ipsilateral cortex on D3 compared to sham-injured rats (t = 2.659, *p* = 0.016; Fig. 4a). There were no significant differences between mFPI and shams on levels of NAA+NAAG, Glu+Gln, GPC+PCh, GSH, or Cr+PCr (all *p* *> *0.05; Fig. 4b–f).

### mFPI induces transient DTI abnormalities

**Figure 5:**
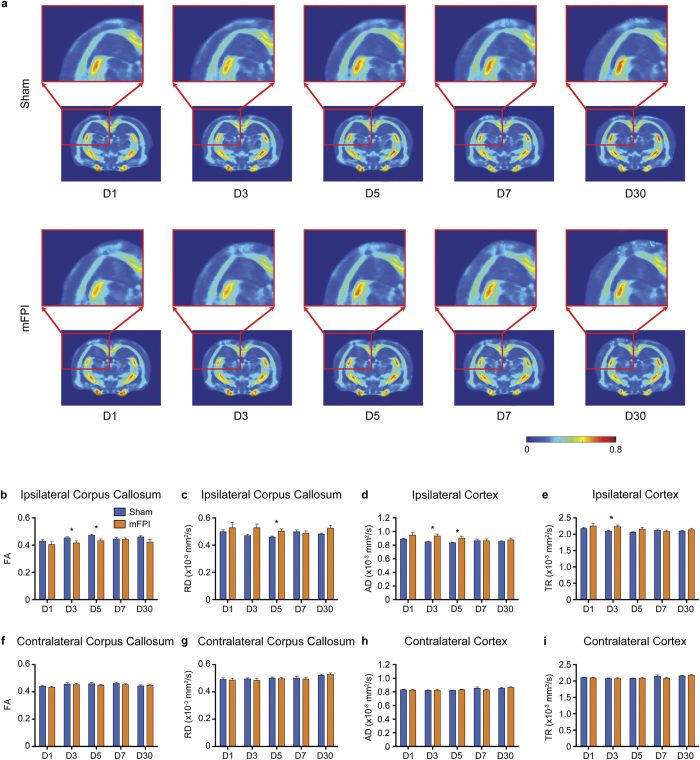
mFPI induces transient DTI abnormalities. (**a**) FA template images for sham (top row) and mFPI (bottom row) rats at each time point. A section of the ipsilateral hemisphere that includes the cortex, corpus callosum and hippocampus is shown enlarged (red box). Qualitatively there appears to be reduced FA in the ipsilateral corpus callosum that persists from D1 to D30. However, ROI-based quantification revealed that mFPI only induced statistically significant reductions in FA at D3 and D5 in the ipsilateral corpus callosum (**b**) with a corresponding increase in RD at D5 (**c**). Additionally, mFPI rats had increased AD at D3 and D5 (**d**) and increased TR on D3 (**e**) in the ipsilateral cortex. There were no differences in any DTI measure in the contralateral hemisphere (**f–i**). Mean ± s.e.m., *mFPI group significantly different than sham group, *p* < 0.05.

ROI-based DTI analyses revealed that mFPI rats had significantly decreased FA in the ipsilateral corpus callosum on D3 (t = 2.240, *p* = 0.038) and D5 (t = 2.620, *p* = 0.017) when compared to shams (Fig. 5b). Additionally, mFPI rats had significantly increased RD in the ipsilateral corpus callosum on D5 (t = 2.462, *p* = 0.024, Fig. 5c); increased AD in the ipsilateral cortex on D3 (t = 2.898, *p* = 0.009, Fig. 5d) and D5 (t = 2.354, *p* = 0.030, Fig. 5d); and increased TR in the ipsilateral cortex on D3 (t = 2.547 *p* = 0.020, Fig. 5e) compared to sham-injured rats. There were no significant differences in any DTI measure in the contralateral hemisphere (Fig. 5f–i).

### mFPI induces persistent tractogram abnormalities

**Figure 6:**
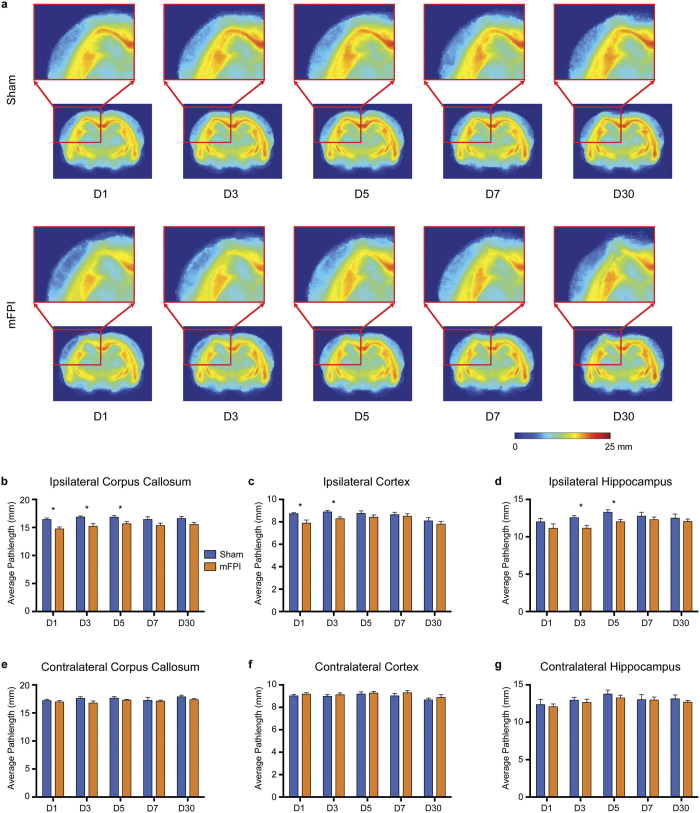
mFPI induces transient reductions in average streamline pathlength. (**a**) Average pathlength template images for sham (top row) and mFPI (bottom row) rats at each time point. Rats given an mFPI had shorter streamlines in the ipsilateral corpus callosum at D1, D3 and D5 (**b**), in the ipsilateral cortex at D1 and D3 (**c**), and in the ipsilateral hippocampus at D3 and D5 (**d**). No differences were observed contralaterally (**e–g**). Mean ± s.e.m., *mFPI group significantly different than sham group, *p* < 0.05.

As shown in Fig. 6, *a-priori* ROI-based analyses revealed that mFPI rats had significantly shorter average pathlength in the ipsilateral corpus callosum on D1 (t = 3.744, *p* = 0.002), D3 (t = 3.204, *p* = 0.005) and D5 (t = 2.548, *p* = 0.020) than their sham-injured counterparts (Fig. 6b). Additionally, rats given an mFPI also had significantly shorter streamlines in the ipsilateral cortex on D1 (t = 2.868, *p* = 0.010, Fig. 6c) and D3 (t = 2.902, *p* = 0.010, Fig. 6c) and in the ipsilateral hippocampus on D3 (t = 2.897, *p* = 0.010, Fig. 6d) and D5 (t = 2.765, *p* = 0.013, Fig. 6d) compared to shams. No significant differences were observed in the contralateral hemisphere at any time point (Fig. 6e–g).

**Figure 7:**
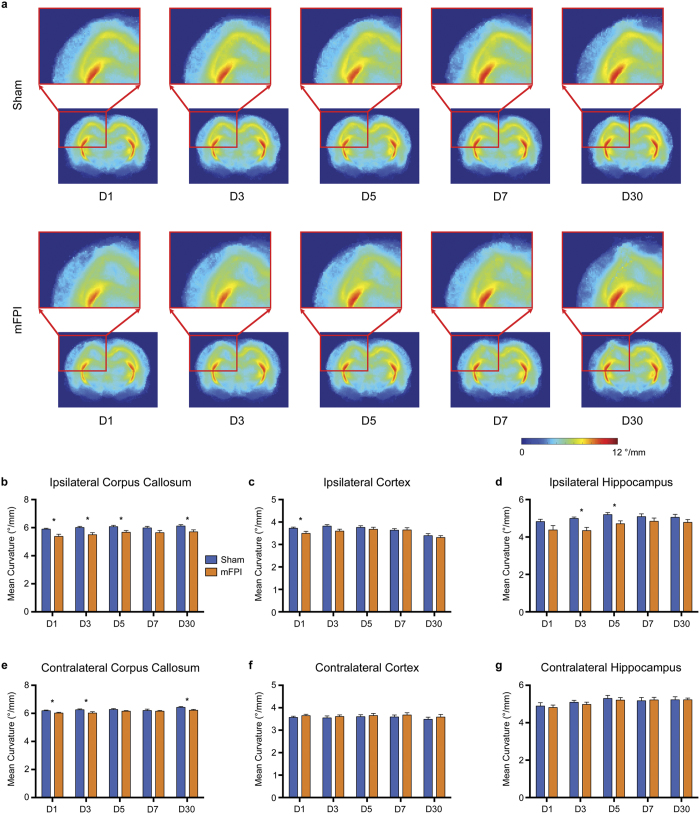
mFPI induces persistent reductions in average streamline curvature. (**a**) TWI-MC template images for sham (top row) and mFPI (bottom row) rats at each time point. Rats given an mFPI had significantly reduced curvature in the ipsilateral (**b**) and contralateral (**e**) corpus callosum that persisted to D30 post-injury. Additionally, mFPI rats had significantly reduced mean curvature in the ipsilateral cortex at D1 (**c**) and the ipsilateral hippocampus on D3 and D5 (**d**). Mean ± s.e.m., *mFPI group significantly different than sham group, *p* < 0.05.

Rats given an mFPI also had significantly reduced mean curvature in the ipsilateral corpus callosum on D1 (t = 3.246, *p* = 0.005, Fig. 7b), D3 (t = 2.994, *p* = 0.008, Fig. 7b), D5 (t = 2.491, *p* = 0.023, Fig. 7b) and D30 (t = 2.397, *p* = 0.028, Fig. 7b), and the contralateral corpus callosum on D1 (t = 2.439, *p* = 0.025), D3 (t = 2.144, *p* = 0.031) and D30 (t = 2.346, *p* = 0.046) compared to sham-injured rats (Fig. 7e). Additionally, rats given an mFPI also had significantly reduced mean curvature in the ipsilateral cortex on D1 (t = 2.156, *p* = 0.045, Fig. 7c) and in the ipsilateral hippocampus on D3 (t = 3.525, *p* = 0.002, Fig. 7d) and D5 (t = 2.590, *p* = 0.019, Fig. 7d).

### mFPI alters the levels of select protein biomarkers in the plasma

**Figure 8:**
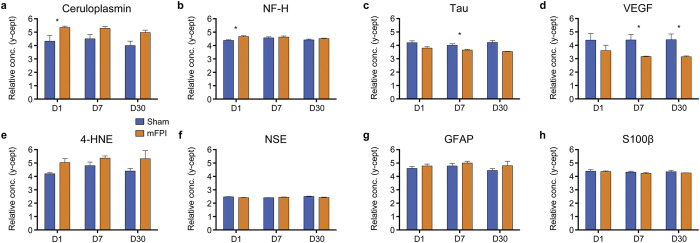
mFPI alters protein biomarker levels in plasma. Rats given an mFPI had significantly increased levels of ceruloplasmin (**a**) and NF-H (**b**) in plasma on D1 compared to sham-injured rats. Additionally, mFPI rats had significantly decreased levels of tau on D7 (**c**), and VEGF on D7 and D30 (**d**) compared to their sham counterparts. There were no statistically significant differences between the groups on any of the other plasma biomarkers (**e–h**). Mean ± s.e.m., *mFPI group significantly different than sham group, *p* < 0.05.

Rats given an mFPI had significantly elevated levels of ceruloplasmin (t = 2.617, *p* = 0.047, Fig. 8a) and NF-H (t = 3.435, *p* = 0.006, Fig. 8b) on D1 compared to sham rats. In addition, mFPI rats had decreased levels of tau on D7 (t = 2.509, *p* = 0.029, Fig. 8c), and decreased VEGF on D7 (t = 3.186, *p* = 0.006, Fig. 8d) and D30 (t = 2.407, *p* = 0.037, Fig. 8d). There were no statistically significant differences in 4-HNE, NSE, GFAP, or S100β plasma levels (all *p* *> *0.05; Fig. 8e–h).

## Discussion

This study employed the rat mFPI model in conjunction with advanced MRI, blood proteomics, and behavioral analyses to assess the ability of these different measures to detect changes after experimental mTBI. We report that an experimental mTBI resulted in transient cognitive abnormalities that normalized by D5. Numerous MRI and blood-based outcomes detected changes beyond the resolution of behavioral symptoms, and in some cases these changes were present at D30. Although all three of the biomarker platforms assessed were able to detect abnormalities after experimental mTBI, our findings suggest that advanced MRI and blood-based proteomics are more sensitive in identifying the duration of mTBI-induced molecular and fine structural changes than the behavioural measures examined in this study.

### Nature of behavioural findings

Transient cognitive deficits were observed in the mFPI group, with no evidence of deficits by D5. This is consistent with previously published findings with the mFPI model^29^,^30^,^31^,^41^, as well as clinical concussion where post-concussive symptoms typically resolve within seven days of injury^4^. Although no group differences were observed on D1, this was likely due to the sham animals requiring some exposure to the water maze before they learned how to complete the task. Because there was no group difference on the measure of swim speed, or on other motor tasks, it is likely that the increased search times and fewer direct and circle swims performed by the mFPI rats were indicative of cognitive impairment and not confounded by motor deficits. Although we found no evidence for behavioral impairments beyond D3, it is important to consider whether other behavioral tasks that were not used in this study may have revealed deficits at other post-injury time points.

Interestingly, there were a number of abnormalities indicated by MRI that were present at the time of the cognitive deficits in mFPI rats. ^1^H-MRS revealed a significant decrease in Ins that corresponded with the onset and resolution of cognitive deficits. As discussed in greater detail below, changes in Ins may reflect an astroglial response, and astrogliosis has previously been associated with cognitive impairments in the days following mFPI^29^. DTI identified abnormalities in the corpus callosum present at D3 indicative of axonal injury, and previous findings have also associated DTI-detected corpus callosum injury with cognitive deficits after mTBI^14^,^15^.

### The biological underpinnings of MRI and blood-based biomarkers

Consistent with neuroimaging results in clinical concussion where conventional structural MRI sequences typically report negative findings^4^, here we found no evidence of overt structural brain damage after mFPI on conventional T_2_^*^-weighted images. However, ^1^H-MRS and DWI were both able to identify mFPI-induced abnormalities. ^1^H-MRS revealed a significant decrease in Ins in mFPI rats at D3, however there were no statistically significant differences between the groups on ^1^H-MRS measures from D5 onwards. Ins is a molecule mostly located within astrocytes and is thus widely considered an astroglia marker^42^. Consistent with our finding here, Ins has previously been found to be decreased in other rat models of TBI^43^, in the motor cortex and hippocampus after sports-related concussion^18^, and in other forms of TBI^44^. It has been speculated that decreased Ins early after TBI may be indicative of cell damage and / or death, or be a consequence of an efflux of Ins from astrocytes in order to regulate volume under conditions of edema^42^. However, it should also be noted that other studies have found increased Ins levels after TBI^45^. The exact reasons for these contradictory findings regarding Ins levels after TBI are not yet known and require further investigation, though temporal and spatial aspects as well as the severity of the injury are all critical factors to consider.

Significant abnormalities in the corpus callosum and cortex of mFPI rats were detected by DTI measures on D3, with some of these changes persisting after cognitive symptoms had normalized. In the ipsilateral corpus callosum there was a significant decrease in FA on D3 and D5, and an increase in RD on D5, in rats given an mFPI. Whereas in the ipsilatereal cortex there was increased AD on D3 and D5, as well as increased TR on D3. Traditional DTI measures, such as FA, AD, RD, and TR may reflect various pathological events that occur after brain injury, including axonal injury, edema, gliosis, and blood barrier disruption, as each of these processes are capable of affecting the diffusivity of water molecules in the brain^46^,^47^. Decreased FA in the corpus callosum has previously been reported after mTBI^15^,^18^,^48^,^49^, and this is widely considered a marker for axonal injury within this major white matter tract. The finding of increased AD in the injured cortex after TBI is consistent with our previous findings^50^ and may be related to gliosis^46^.

Track-weighted imaging methods are relatively new. Derived from properties of the streamlines, such as length, curvature and density, these maps give additional contrasts that may be sensitive to the subtle changes occurring in the brain after mTBI. Here, we found that the average pathlength was reduced in the ipsilateral corpus callosum to D5. This is consistent with the original average pathlength article of Pannek and colleagues who reported a significant decrease in pathlength in the corpus callosum of a TBI patient compared to a cohort of controls^36^. Additionally, we also observed reduced average pathlength in the ipsilateral hippocampus to D5 and in the ipsilateral cortex to D3. These changes likely reflect underlying DTI changes within these regions, which would be expected to cause the premature termination of streamlines. While the average pathlength did not appear to be any more sensitive than traditional DTI measures, we found changes in streamline curvature at D30 in both the ipsilateral and contralateral corpus callosum, suggesting that streamline curvature may be more sensitive to the subtle changes occurring in the brain after mFPI. Reduced streamline curvature may also reflect the early termination of streamlines; however, further research is required to determine how changes in streamline properties, such as length and curvature, relate to the underlying pathology. Additionally, as we ceased this study at D30 it remains to be seen whether these changes are permanent, or whether there is still the potential for full recovery.

RPPM analysis of blood-based protein biomarkers revealed that mFPI resulted in increased levels of ceruloplasmin and NF-H at D1, decreased levels of tau at D7, and decreased levels of VEGF at D7 and D30. Ceruloplasmin is considered a marker for metabolic abnormalities post-TBI^19^, and our finding of elevated circulating ceruloplasmin at D1 is consistent with previous studies that also report increased ceruloplasmin in the early stages post-TBI^26^. Ceruloplasmin, a positive acute-phase protein, is involved in exporting free iron and in iron metabolism in the brain^26^,^51^. TBI causes elevated levels of iron, which can trigger oxidative stress and neuroinflammation^26^,^51^. Thus, the increased ceruloplasmin levels observed in this study may be in response to an mFPI-induced acute increase in free iron.

Increased levels of circulating NF-H have been reported after TBI^20^,^52^,^53^, including mTBI^26^, and is considered a marker of axonal injury. Consistent with previous findings, the current study also found elevated levels of NF-H in the plasma of mFPI rats at D1. Combined, the DWI and NF-H findings support the notion that mFPI induced axonal injury. Though it is interesting to note that the plasma changes in NF-H manifested earlier (i.e., D1) but were not present at the later time points, whereas DWI changes emerged later (i.e., D3) and were present at D30.

Tau was decreased in the plasma of mFPI rats at D7, which is consistent with previous studies from our laboratory indicating decreased levels of total tau in brain tissue following TBI^54^. However, our plasma findings contradict those from other studies that have reported increased levels of circulating tau, which is postulated to be a marker of axonal injury^19^, after mTBI^25^,^26^. The reasons for these discrepancies should be further explored, and the mechanism/pattern of injury (i.e., blast versus focal versus diffuse) may be an important factor to consider as this could affect the consequent pathophysiological cascade. Unfortunately, the absence of a specific phospho-tau antibody suitable for RPPM at the time of the study prevented us from determining the effect of the injury on the ratio between total tau and phosphorylated tau. In our earlier studies, we have found increased levels of phosphorylated tau, an important cofactor in neurodegenerative diseases such as Alzheimer’s and CTE, in brain tissue following TBI^54^,^55^. It is possible that the reduced circulating tau in this study may be related to an increase in phosphorylated-tau. Because tau abnormalities are implicated in the cumulative and long-term effects of mTBIs, it is important that further studies are conducted to gain a more comprehensive understanding of the nature of these changes in the brain, how they relate to other pathology (e.g., axonal injury, neuroinflammation), and how blood-protein levels reflect these changes.

VEGF is known to be a potent stimulator of angiogenesis (i.e., the formation of new blood vessels), but VEGF also has direct effects on other types of neural cells^56^. In fact, inhibition of VEGF signaling results in increased cell death after TBI^57^. Conversely, treatment with VEGF has been found to stimulate neurogenesis and is neuroprotective after experimental TBI in rats^58^,^59^. In this study, VEGF was decreased at D7 and D30 in the plasma of rats given an mFPI. These findings may have important implications, particularly with regards to susceptibility to repeated mTBI and neurodegeneration. For example, angiogenesis post-TBI may be an important recovery process that restores adequate blood and oxygen supply to damaged tissue^60^. If this process has been compromised in rats given an mFPI, the injured tissue may be at increased vulnerability if exposed to a consequent insult. Notably, reduced VEGF levels have been shown to cause neurodegeneration by impairing neural tissue perfusion^56^, and mice with reduced VEGF levels develop motor neuron degeneration reminiscent of amyotrophic lateral sclerosis^61^, a neurodegenerative disease that has been epidemiologically linked to brain trauma^8^. Taken together, this novel finding of decreased VEGF in the sub-acute to long-term stages after a single mTBI may have important implications in understanding the underlying mechanisms involved in ICV, the degenerative effects of repeated mTBI, as well as potential treatment strategies, and should be further investigated in future studies.

Although other studies have reported that mTBI induces changes in S100β, GFAP, 4-HNE, and NSE^24^,^25^,^26^,^62^,^63^, we did not find any statistically significant differences pertaining to these markers in our study. Variable findings between laboratories/studies with regards to specific blood-based protein biomarkers is not uncommon and may be related to methodological differences^24^. Our findings also indicate temporal complexities with the various markers, which is consistent with previous findings suggesting that a single point assay of a single protein biomarker is not able to capture the entire pathophysiological cascade of mTBI^23^,^26^. However, when taken together, RPPM analysis of plasma proteins did reveal mFPI-induced changes that emerged on D1 (i.e., before significant behavioral or MRI-related changes), and were present at D7 and D30 (i.e., after behavioral symptoms normalized).

### Future direction and hurdles

While the use of symptom-based tools can aid in mTBI diagnosis, their subjective nature, limited sensitivity, need for accurate baseline measures, practice effects, and lack of any pathophysiology quantification are problematic^4^,^64^,^65^,^66^. It is for these reasons that objective biomarker platforms capable of assessing biological changes are being pursued to either supplement or replace symptom-based management approaches. This study provides evidence that DTI and tractography methods detect changes for up to 30 days post-injury, long after behavioral changes had normalized, and should continue to be developed and employed in future investigations of mTBI. In particular, clinical studies with serial neuroimaging after mTBI are required to better characterize the temporal progression of injury and recovery, and how these changes relate to symptoms and neuropsychological alterations. Unfortunately, due to the high cost, need for specialized equipment/personnel, and the additional research required validating their use as reliable and effective biomarkers, it is unlikely that neuroimaging will be widely accessible as a clinical mTBI management tool for routine clinical use in the near future.

Alternatively, blood-based biomarkers may represent a more readily available strategy to provide information about ongoing pathophysiological changes after mTBI that aids in diagnosis and monitoring injury progression/recovery^24^. In this study tau and VEGF detected changes that were present after behavioral recovery, and there are other protein biomarkers that may be sensitive to the neuronal and glial cell loss, metabolic abnormalities, neuroinflammation, axonal injury, and other pathophysiological changes associated with mTBI^19^,^24^. In addition, microRNAs represent another source of promising blood-based biomarkers for mTBI that should continue to be explored^67^. However, to our knowledge there are no detailed longitudinal studies that have assessed blood biomarkers in mTBI patients in order to validate their use as diagnostic and prognostic tools^24^. Furthermore, the lack of standardized methods to quantify blood biomarkers, and the need for baseline measures for each individual and/or validated cut-off points are additional limitations to be addressed before the use of blood-based biomarkers in mTBI management^24^.

While MRI and blood-based biomarker platforms may have the capacity to help guide medical decisions, to date, there is no direct evidence that the implementation of any of these methods will actually prevent or reduce the long-term effects of repeated mTBIs. Because the studies required to assess this would take decades to complete in humans, and would be confounded by a number of factors, animal model studies that administer repeated mTBIs with the inter-injury times guided by a specific biomarker would be useful in providing evidence that management based on particular biomarkers is effective.

There are some limitations with this study that should also be considered. The mFPI requires a craniotomy and the administration of anesthetic, both of which are not typically present in the clinical mTBI setting and have the potential to confound results^68^,^69^,^70^. Nonetheless, previous studies have found that a single mFPI can result in transient motor and cognitive deficits^29^,^31^,^41^,^71^, as well as abnormalities in other behaviors^29^, sleep^72^, and electrophysiology^73^,^74^. An mFPI also induces transient neuroinflammation^29^,^30^, axonal injury^29^, the hyperphosphorylation of tau^55^, and reduced cerebral blood flow^73^, but does not result in significant neuronal loss, visible brain contusion, or focal lesion^29^,^30^,^31^,^71^,^73^. These changes are consistent with those that might occur in clinical mTBI. Taken together, the use of the mFPI to model mTBI in this and future studies is warranted, particularly while newer models of mTBI/concussion that avoid limitations such as craniotomy and anesthetic are developed, characterized, validated, and become widely available to other laboratories. Another limitation is the lack of histological/immunohistochemical analyses of brain tissue that may have complemented the other outcomes. For example, additional information pertaining to the nature of the brain damage and other neuropathological changes observed in rats given an mFPI (e.g., confirming DTI findings of axonal injury in the corpus callosum; relationship between Ins and neuroinflammation; localization of blood biomarker abnormalities such as VEGF) would have been informative and should be examined in future studies. Though it is worth noting that previous studies of neuropathology after an mFPI in the rat have found that it causes increased silver staining and amyloid precursor protein immunoreactivity (i.e., markers of axonal injury) in the corpus callosum and other white matter tracts, as well as neuroinflammation in the frontal and parietal cortex, corpus callosum, and thalamus^29^,^31^, all of which may be of relevance to the current findings.

## Conclusion

This study investigated the use of MRI, blood proteomics, and behavioral methods as markers to detect changes and estimate recovery after experimental mTBI. All three of the platforms were able to detect abnormalities after mFPI; however, advanced MRI and blood-based proteomics proved to be more sensitive for the detection of persisting and/or long-term changes than the behavioral measures examined. In particular, our findings related to blood biomarkers, such as VEGF and tau, might have important implications in the context of ICV and the cumulative/chronic consequences of repeated mTBI. Furthermore, to our knowledge this is the first study to report that advanced track-weighted imaging measures, such as mean curvature and average pathlength, are able to detect mTBI and may be more sensitive to persisting white matter abnormalities than traditional DTI measures (e.g., FA). It is important to note that further studies are required to determine the biological significance/underpinnings of these markers and their relationship to neurobehavioral changes, and there are a number of challenging hurdles before these markers can be reliably applied in the clinical mTBI setting. Nonetheless, the characterization and utilization of these and other objective biomarkers should continue to be pursued in both the laboratory and the clinic due to their potential value in guiding management decisions, including “return to play”, following concussion.

## Additional Information

**How to cite this article**: Wright, D. K. *et al*. Behavioral, blood, and magnetic resonance imaging biomarkers of experimental mild traumatic brain injury. *Sci. Rep.* **6**, 28713; doi: 10.1038/srep28713 (2016).
